# Dementia Data Landscape 1. Cohorts

**DOI:** 10.1002/alz.70901

**Published:** 2025-11-21

**Authors:** Amelia Morgan, Conor Durkin, Benjamin Tari, Emma Squires, Simon Young, Joshua Bauermeister, Sarah Bauermeister, John Gallacher

**Affiliations:** ^1^ Dementias Platform UK Department of Psychiatry University of Oxford Oxford UK; ^2^ Swansea University Medical School Swansea University Swansea UK

**Keywords:** Alzheimer's disease, clinical cohort, cohort study, C‐Surv, data access, data characterization, data landscape, data repurpose, data reuse, dataset discovery, dementia, metadata, population cohort

## Abstract

**INTRODUCTION:**

Understanding and maximizing complex health data is crucial for accelerating discovery, translational research, funding priorities, and improving data management. Rapid, cost‐effective progress can be made by repurposing datasets. This work explores the dementia cohort landscape, identifies cohorts relevant to dementia translation, and highlights areas to strengthen health cohort infrastructure.

**METHOD:**

PubMed was searched for publications utilizing dementia‐related cohorts (1970–2024), supplemented by international dementia data platforms. A template aligned with the C‐Surv data model was used to summarize administrative details and the presence of measurements across 17 themes.

**RESULTS:**

From 4596 publications and 11 data platforms, 883 cohorts were identified (558 population and 325 clinical). Of these, 74% indicated data availability for future research, though metadata reporting varied. Cohort metadata are accessible via the landscape tool.

**DISCUSSION:**

This work reveals extensive global dementia‐related data for repurposing and identifies priority areas for improvement, including metadata transparency, data accessibility, and locations to prioritize for future research.

**Highlights:**

- A total of 883 cohorts were identified globally (1970 to 2024): 558 population and 325 clinical
- The Global South is substantially underrepresented
- Seventy‐four percent of cohorts offer data access, but protocols and metadata quality vary widely
- Only 45% of cohorts were discoverable via existing data platforms
- The online landscape tool enables strategic discovery and reuse of dementia data

## 1 BACKGROUND

Cohorts, defined as the serial assessment of defined human populations over time, provide valuable insights into the etiology and progression of disease. They inform etiology at the molecular, cellular, behavioral, and social levels, as well as the progression from pre‐clinical risk through diagnosis, treatment, and social care. Cohorts large enough to enable stratified analysis inform mechanism discovery and precision approaches to treatment. Although cohort research‐cycle times are protracted, a cohort's scientific value accrues over time and, from a strategic perspective, provides high‐value scientific returns per unit investment. Recently, the potential value of cohorts has increased as enhancement by linkage to administrative health and social care data becomes available.

The dementia cohort data landscape is fragmented. This is due in part to many cohorts being designed primarily to investigate cardiovascular, metabolic, and cancer‐related disorders, or aging in general, with neurodegeneration being a secondary outcome. By design, population cohorts are relevant to multiple outcomes, such that a cohort focusing on heart disease at inception may become increasingly relevant to dementia over time. Only recently has dementia become the primary focus of cohort studies.

In addition, cohort data are complex and diverse. Although understanding of molecular and cellular mechanisms is non‐trivial, the measurement of clinical and behavioural phenotypes and environments is highly variable across and within longitudinal studies. This highlights the value of data, metadata, and reporting standards in making cohort data more easily discoverable and accessible.

Finally, search engines are optimized to identify findings and authors, not datasets. It remains labor intensive for researchers to trawl through multiple reports to identify independent datasets of potential interest. These reports rightly describe details of the study that are immediately relevant to the research question, rather than the broader context of the dataset.

This article provides an overview of the cohort data landscape. We: (1) investigate the relative importance of cohort designs for dementia research, (2) characterize dementia‐relevant cohorts, and (3) develop a dementia‐focused data landscape discovery tool.

**RESEARCH IN CONTEXT**

1. **Systematic review**: We reviewed the literature using PubMed, revealing that although many dementia cohort studies exist, these have not been mapped systematically on a global scale in terms of metadata quality, discoverability, and data access protocols. Our research fills this gap by providing a comprehensive catalogue of dementia cohorts, focusing on improving data accessibility and global representation.
2. **Interpretation**: This study presents the most extensive mapping of global dementia cohort datasets to date, covering over 880 independent studies. Although data availability is increasing, gaps remain in metadata completeness, geographical representation, and accessibility. Our findings uncover opportunities for data repurposing and highlight barriers to equitable data use.
3. **Future directions**: Future efforts should prioritize improved metadata reporting, streamlined access, and greater inclusivity in underrepresented regions, especially the Global South. International initiatives to standardize cohort documentation and enable secure data access are crucial for maximizing the potential of dementia data.

## 2 METHODS

### 2.1 The relative importance of cohort data for dementia (publications survey)

The following search terms were used to interrogate PubMed for publications using human data (i.e., most relevant to the translation of discoveries into treatments).

- ***All publications***: *(((“dementia”[Title] OR “alzheimer”[Title] OR “alzheimer's”[Title] OR “parkinson”[Title] OR “parkinson's”[Title] OR “PD”[Title] OR “lewy body”[Title] OR “LBD”[Title] OR “FTD”[Title] OR “cognitive impairment”[Title] OR “MCI”[Title] OR “cognitive decline”[Title]) NOT “review”[Title/Abstract]) AND 1980/01/01:2024/12/31[Date—Publication])*
- ***Cohorts***: *(((“dementia”[Title] OR “alzheimer”[Title] OR “alzheimer's”[Title] OR “parkinson”[Title] OR “parkinson's”[Title] OR “PD”[Title] OR “lewy body”[Title] OR “LBD”[Title] OR “FTD”[Title] OR “cognitive impairment”[Title] OR “MCI”[Title] OR “cognitive decline”[Title]) NOT “review”[Title/Abstract]) AND ((cohort[Title]) OR (prospective[Title])) AND 1980/01/01:2024/12/31[Date—Publication])*
- ***Trials***: *(((“dementia”[Title] OR “alzheimer”[Title] OR “alzheimer's”[Title] OR “parkinson”[Title] OR “parkinson's”[Title] OR “PD”[Title] OR “lewy body”[Title] OR “LBD”[Title] OR “FTD”[Title] OR “cognitive impairment”[Title] OR “MCI”[Title] OR “cognitive decline”[Title]) NOT “review”[Title/Abstract]) AND (trial[Title]) AND 1980/01/01:2024/12/31[Date—Publication])*
- ***Registers***: *(((“dementia”[Title] OR “alzheimer”[Title] OR “alzheimer's”[Title] OR “parkinson”[Title] OR “parkinson's”[Title] OR “PD”[Title] OR “lewy body”[Title] OR “LBD”[Title] OR “FTD”[Title] OR “cognitive impairment”[Title] OR “MCI”[Title] OR “cognitive decline”[Title]) NOT “review”[Title/Abstract]) AND ((register[Title]) OR (registry[Title])) AND 1970/01/01:2024/12/31[Date—Publication])*

Each calendar year 1970–2024 (inclusive) was searched in turn. A broad search strategy was employed to capture all publications that reference or utilize a relevant cohort, aiming to maximize the identification of cohort studies. Results were limited to English‐language abstracts and articles in open‐access, peer‐reviewed journals. Results were extracted from PubMed and uploaded to Rayyan for further analysis.^1^ In Rayyan, publication titles and abstracts were reviewed to identify the dataset(s) utilized in each publication and to categorize cohort‐related publications based on type of cohort studied. We separate clinical and population cohorts as being differentially informative. Population cohorts were defined as following a general population sample, such as the National Institutes of Health (NIH) All of US Research Program, the Rush Memory and Aging project (ROSMAP), and UK Biobank.^2^, ^3^, ^4^ Clinical cohorts were defined as following a sample selected for a specific disease, often including symptomatic or pre‐symptomatic patients, such as the Genetic Frontotemporal Initiative (GENFI), which investigates individuals with symptoms of frontotemporal dementia (FTD) or at‐risk gene carriers; and determinants and evolution of AlzheiMer's disease and related disorders (MEMENTO), which follows participants recruited in memory clinics with varying cognitive symptoms and subjective cognitive complaints.^5^, ^6^, ^7^

In cases where the abstract and title lacked sufficient detail, the full publication was reviewed. This approach provided an overview of the frequency of cohort‐, register‐, and trial‐related outputs, to illustrate trends in publication counts over time.

### 2.2 Characterizing the cohort data landscape (survey of datasets)

In parallel with Rayyan analysis, dataset names identified from the PubMed cohort‐related search results were compiled to establish a comprehensive list of global cohorts. To ensure accuracy and reliability, a second researcher, blinded to the first reviewer's interpretations, repeated the process independently. Conflicts were resolved by a third reviewer.

Additional datasets were identified from existing international dementia‐related data platforms, including the DPUK Data Portal,^8^ AD Workbench (ADWB),^9^ Maelstrom,^10^ Global Alzheimer's Association Interactive Network (GAAIN),^11^ EU Joint Programme Neurodegeneration Disease Research (JPND),^12^ Critical Path for Alzheimer's Disease Consortium (CPAD),^13^ European Medical Information Framework (EMIF),^14^ and Common Alzheimer's and Related Dementias Research Ontology (CADRO).^15^

The search was limited to cohorts that (1) had an identifiable study name, (2) included participants 18 years of age or older, and (3) had metadata available in the public domain. We excluded datasets focusing on infectious diseases, surgical interventions, or animal studies, as well as administrative databases (including insurance data) and those lacking sufficient metadata—specifically, datasets missing basic administrative details such as the institution, location, study dates, sample size, principal investigator, or study purpose.

A core set of variables was identified to characterize each cohort using the C‐Surv data model.^16^ C‐Surv is optimized for the discovery of research cohort data. It has a four‐tier nested structure to facilitate data selection.[Table alz70901-tbl-0001] Dataset information was sourced from various platform metadata tools, data matrices, cohort directories, data discovery tools, cohort websites, data dictionaries, and publications (e.g., cohort profiles and protocol papers) related to the included cohorts. Cohort information was recorded in the C‐Surv template using Microsoft Excel. Administrative information and some demographic data were recorded in full, whereas the remaining categories were recorded in a binary (yes/no) format to indicate the presence or absence of measurement in each dataset. “Undetermined” was recorded where it was not possible to accurately quantify the presence or absence of a variable, typically due to vague or limited metadata.

Cohort characterization focused on geographical location, sample characteristics, duration of study, follow‐up frequency, availability of data, and access to bio‐sample. Metadata were extracted from the most recent available documentation identified.

Following data entry, basic descriptive statistics (i.e., means, SDs, and frequencies) were calculated directly within Microsoft Excel to summarize the cohorts. Data filtering and sorting functions were applied to segment data based on specific criteria. Additional administrative information, such as research institution/organisation, principal investigator(s), and other relevant details, was also collected. This highlights which data domains are typically well documented versus those that lack clear reporting across datasets.

### 2.3 Data landscape tool

To make these data more easily discoverable, metadata were sourced from various platform metadata tools, data matrices, cohort directories, data discovery tools, cohort websites, data dictionaries, and publications (e.g., cohort profiles and protocol papers) related to the dataset. A core set of variables and design features was identified, optimized for dementia as the primary outcome of interest. Metadata were aligned using C‐Surv as a common data model. Using the C‐Surv data model, 17 data themes were selected as being particularly relevant to dementia (Table 1). The “device‐derived phenotypes” theme was excluded due to its limited representation across the included cohorts. The landscape tool was populated at two levels of granularity. Design characteristics (i.e., age, size, number of waves), were recorded as numeric values, whereas research variables were recorded as present (measured), absent (not measured), or undetermined. To track the progress of each cohort over time, its operational status (i.e., active, closed, etc.) was also recorded. Data were collated in Microsoft Excel, and Microsoft Power BI was used to present this on the DPUK Data Portal website, providing a publicly available interactive tool with search and filter functions.^17^ The tool was iteratively tested for functionality and usability by the research team, with feedback used to refine the interface and ensure accurate representation of cohort metadata.

**TABLE 1 alz70901-tbl-0001:** Landscape tool themes.

C‐Surv data theme	
1	**Administration**: study name, purpose, country, size, age, sex, dates, access
2	**Sociodemographic indicators**: demographic, education, economic
3	**Early life adversity**: early life sociodemographic, childhood environment
4	**Physical measures**: anthropometry
5	**Medical history**
6	**Family disease history**
7	**Psychological status**
8	**Cognitive status**: memory, processing speed, diagnosis, emotional processing
9	**Lifestyle behaviour**: diet, substance use, sleep, physical activity
10	**Life functionality**: activities of daily living
11	**Physical environment**: housing, pollution
12	**Social environment**: critical events, social engagement
13	**Imaging**: brain MRI, PET, other
14	**Linkage data**: electronic health records
15	**Healthcare utilisation**: hospital and GP visits
16	**Biosample collection**: blood, urine, CSF
17	**Molecular assays**: genomics, proteomics, metabolomics

## 3 RESULTS

### 3.1 Survey of publications

**FIGURE 1:**
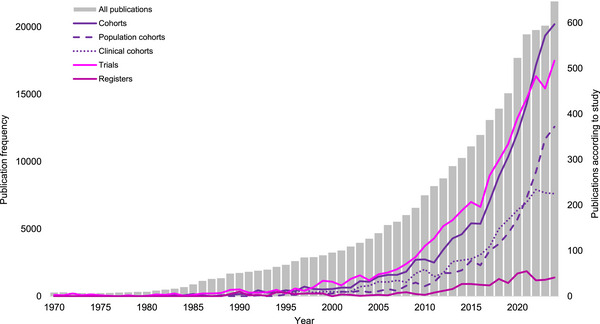
Trend in annual dementia publications. Gray bars show the number of dementia‐related studies published each year from 1970 to 2024 (inclusive). Overlaid colored lines represent the number of these studies classified as cohort studies (population‐based and clinical), clinical trials, and registries.

A total of 290,058 dementia‐related publications using or referring to human data were found. The number of publications increased exponentially from 275 in 1970 to 21,900 in 2024 (Figure 1). Publications utilizing population and clinical cohorts began to increase in the 1990s, from a base of 5 (1990) to 597 (2024). The number of clinical cohorts has increased from 5 to 225 per year over this period, and population cohorts increased from 0 to 372 per year.

In contrast, the number of publications utilizing clinical trial data did not increase materially until the 1980s, with a greater number of publications per year compared to those related to cohorts from 15 (1990) to 517 (2024). It is important to note that this does not necessarily reflect the number of datasets produced; typically, a single cohort will produce more publications than a clinical trial. Of interest, research registers have only shown growth since 2010, possibly reflecting the increasing importance of streamlining trial recruitment for drug development. From these numbers, the scientific value of population and clinical cohort studies is increasingly recognized.

### 3.2 Survey of datasets

**FIGURE 2:**
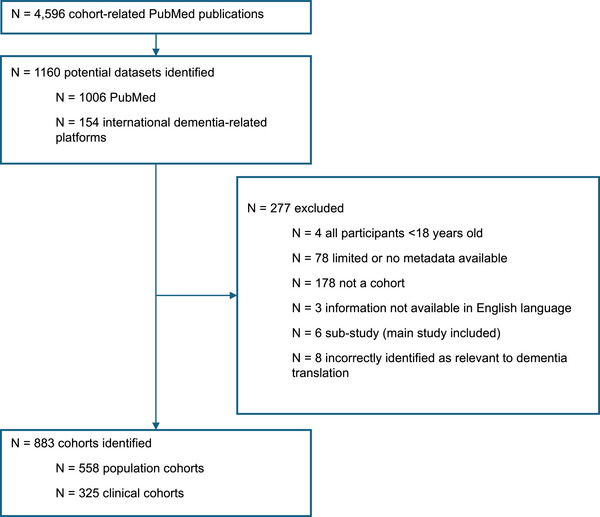
Flow diagram of dataset selection.

Interrogation of the cohort‐related studies revealed a total of 4596 publications related to dementia cohorts (Figure 2). Following review, 1160 independent longitudinal datasets were identified. Of these, 277 were excluded due to limited or no metadata (*n* = 78), failure to satisfy the definition of a cohort study (*n* = 178), inclusion of only participants <18 years of age (*n* = 4), being a sub‐study of another included cohort (*n* = 6), incorrectly identified as relevant to dementia translation (*n* = 8), or information not available in the English language (*n* = 3). In total, 883 cohorts were retained, comprising 558 population cohorts and 325 clinical cohorts.

#### 3.2.1 Geographical distribution

Independent national level datasets were identified in 53 countries, with a further 5 countries (Albania, Bangladesh, Sri Lanka, Cambodia, and Cuba) represented in 25 multinational cohorts (Table 2). At a continental level, there was a stark contrast in the collection of dementia‐relevant cohort data. Europe and North America generate the vast majority of cohort data. The Global South, however, is severely underrepresented, particularly Africa, South America, and Southeast Asia.

**TABLE 2 alz70901-tbl-0002:** Cohorts according to location.

Continent	Country	Number of population cohorts	Number of clinical cohorts	Total number of participants	Participants/per head of population (%)	Data availability (%)
**Africa**	Central African Republic	1	0	2002	0.04	100.00
	Nigeria	1	0	2149	<0.01	100.00
	South Africa	2	0	236,238	0.37	50.00
	**Africa Total**	4	0	240,389	n/a	75.00
**Americas**	Argentina	0	1	56	<0.01	100.00
	Brazil	7	1	41,523	0.02	50.00
	Canada	18	9	859,100	2.16	74.07
	Chile	2	0	11,232	0.06	100.00
	Colombia	0	1	1784	<0.01	0.00
	Costa Rica	1	0	2827	0.06	100.00
	Dominican Republic	0	1	925	0.01	100.00
	Ecuador	2	0	1446	0.01	50.00
	Mexico	2	0	176,295	0.13	100.00
	USA	116	64	6,046,923	1.75	69.83
	Venezuela	1	0	3657	0.01	0.00
	International	1	0	3655	n/a	100.00
	**Americas Total**	150	77	7,149,423	0.71	69.91
**Asia**	China	37	11	1,963,542	0.14	60.42
	India	3	1	33,639	<0.01	75.00
	Indonesia	1	0	278	<0.01	100.00
	Iran	4	0	247,462	0.27	100.00
	Israel	2	2	187,197	1.99	75.00
	Japan	29	9	1,028,839	0.83	50.00
	Korea	13	4	218,669	0.42	52.94
	Malaysia	2	1	110,210	0.31	100.00
	Pakistan	1	0	150,000	0.06	100.00
	Philippines	2	0	4694	<0.01	100.00
	Qatar	1	0	15,000	0.49	100.00
	Saudi Arabia	2	0	102,000	0.30	100.00
	Singapore	8	3	101,763	1.74	54.55
	Taiwan	9	5	148,148	0.64	92.86
	Thailand	3	0	162,480	0.23	66.67
	United Arab Emirates	0	1	50	<0.01	100.00
	Vietnam	1	0	1200	<0.01	100.00
	International	2	0	310,000	n/a	100.00
	**Asia Total**	120	37	4,785,171	0.10	64.97
**Europe**	Austria	2	0	180,606	1.98	100.00
	Belgium	2	2	68,830	0.59	100.00
	Czechia	0	1	2155	0.02	100.00
	Denmark	14	5	468,530	7.84	57.89
	Estonia	2	0	400,000	29.4	100.00
	Finland	22	5	1,456,950	25.94	81.48
	France	19	20	886,348	1.33	61.54
	Germany	16	21	614,657	0.73	86.49
	Greece	4	2	34,987	0.35	50.00
	Hungary	1	0	8000	0.08	0.00
	Ireland	1	1	8760	0.17	66.67
	Italy	11	14	121,911	0.21	68.00
	Luxembourg	1	1	2086	0.31	100.00
	Netherlands	22	27	1,505,750	8.26	79.59
	Norway	9	10	503,609	9.03	77.78
	Portugal	1	1	2947	0.03	50.00
	Spain	7	17	83,576	0.17	66.67
	Sweden	29	5	1,657,007	15.62	91.48
	Switzerland	4	5	37,458	0.42	77.78
	UK	86	50	4,652,308	6.72	91.11
	International	3	5	25,104	n/a	71.43
	**Europe Total**	256	192	12,721,579	1.71	80.31
**Oceania**	Australia	20	9	1,165,022	4.36	75.86
	New Zealand	2	1	1062	0.02	100.00
	**Oceania Total**	22	10	1,166,084	n/a	78.13
**Multicontinental**		6	9	316,092	n/a	57.14
**Total**		558	325	26,378,738	0.32	73.61

#### 3.2.2 Start dates

**FIGURE 3:**
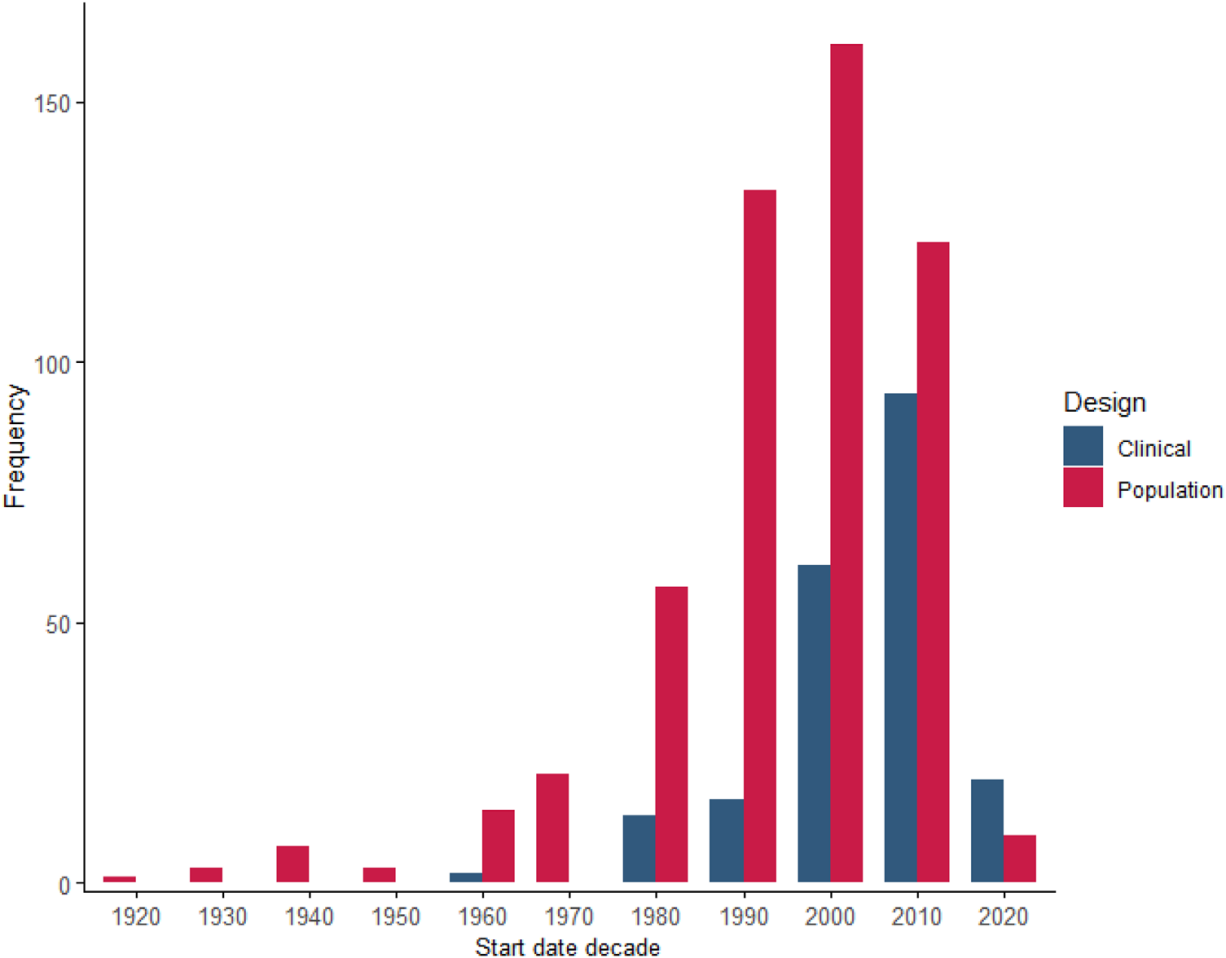
**Cohorts according to start decade**. Number of cohorts according to the decade of study commencement. Dichotomized by study design: clinical cohorts (blue) and population cohorts (red).

Examining start dates of cohort studies shows a decade‐on‐decade incremental increase in population‐based studies leading up to the millennium, after[Table alz70901-tbl-0002] which fewer studies were initiated (Figure 3). Although this may be due to the commissioning of fewer but larger studies over time, this trend has not yet pulled through in terms of overall participant numbers per decade, with most participants being recruited between 2000 and 2020 (n = 13,464,924). However, with the number of at‐scale studies increasing and recruitment ongoing, these figures may be an underestimation of population cohort recruitment.

For clinical cohorts, however, there has been a steady increase since the 1960s. This reflects increased interest in neuropathology in the development of symptomatic and disease‐modifying therapies. Low numbers for both designs in the 2020s may be due to incomplete reporting for this decade, a lag in study registration and publication, or pharmaceutical disinvestment in neurodegeneration prior to the recent licensing of anti‐amyloid therapies.

#### 3.2.3 Sample characteristics

Most cohorts (95.1%) comprised men and women, with roughly half of the remainder recruiting either men (2.6%) or women (2.3%).

**FIGURE 4:**
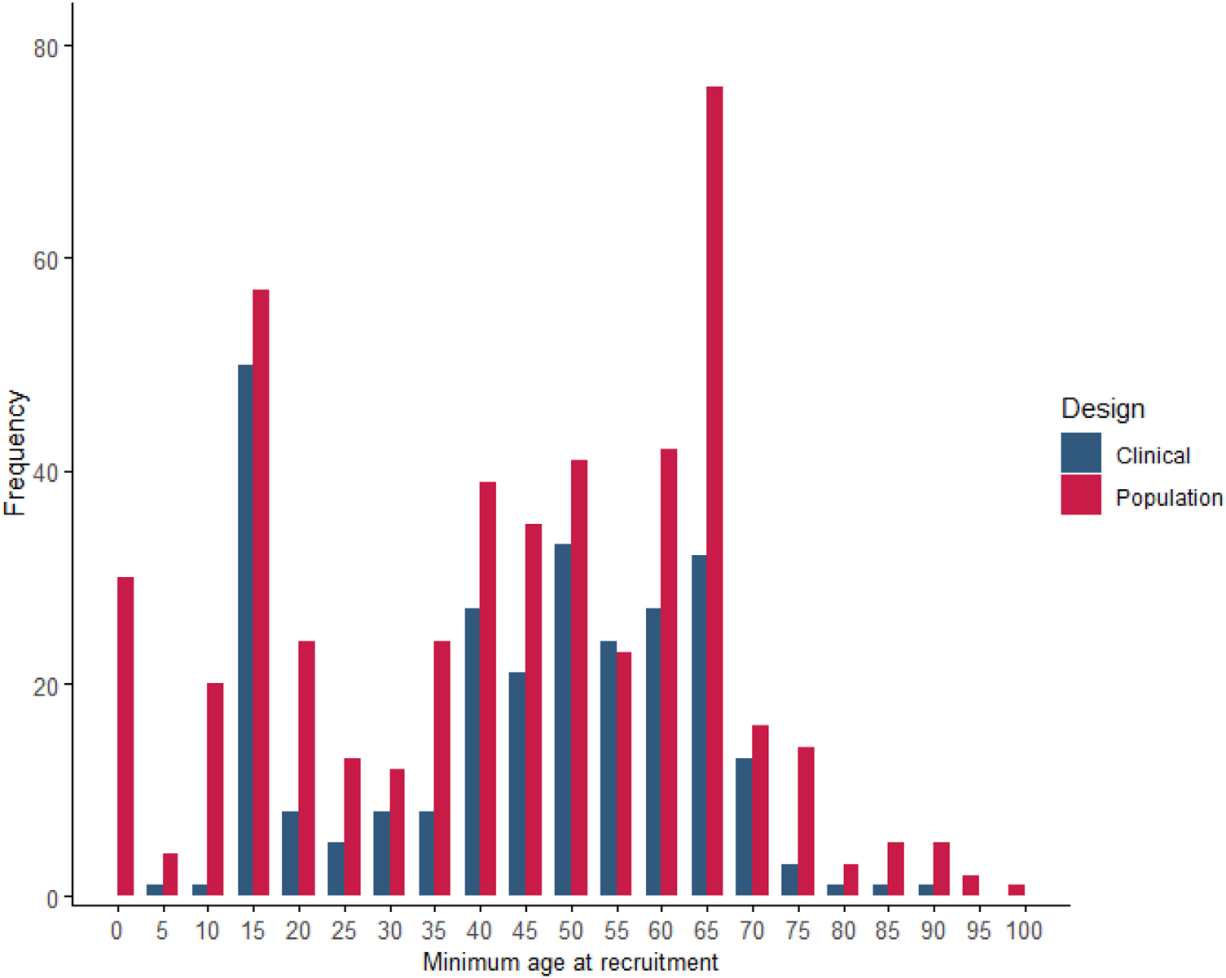
Minimum age at recruitment by cohort design. Minimum age at recruitment according to study design: clinical cohorts (blue) and population cohorts (red).

The distribution of age varied according to design (Figure 4). For population cohorts, 25 birth and early age cohorts are available to provide evidence on early exposures and subsequent dementia risk. A further recruitment spike occurs for adolescent lifestyle and mental health cohorts. Middle‐age recruitment occurred largely for cardiovascular disease, with these cohorts maturing into dementia cohorts over time. From the age of 60, dementia‐ and aging‐specific cohorts begin to be recruited. The slight uptick in later‐life recruitment reflects interest in superagers.

For clinical cohorts, interest begins in adolescence for high‐penetrance genetic disorders. There is most interest at middle age, at which point neuropathology is more frequently diagnosed.

#### 3.2.4 Data Discovery

Of the 883 cohort datasets identified, 395 (45%) could be found using 11 dementia‐relevant discovery platforms (Table 3). Of the remainder, 478 (54%) cohorts were found using PubMed, and an additional 10 (1%) were found on the web. Of the 395 cohorts found on data platforms, 283 (72%) were discoverable only on one of the platforms, suggesting only marginal cross‐platform overlap.

**TABLE 3:** Data platforms according to functionality.

Platform	Discovery	Access brokerage	Repository	Analysis	Number of datasets (%)
**ADWB**	✓	✓	✓	✓	18 (2.04%)
**AMP‐PD**	✓	✗	✗	✗	5 (0.67%)
**DPAU**	✓	✓	✓	✓	23 (2.60%)
**DPUK**	✓	✓	✓	✓	57 (6.46%)
**EMIF**	✓	✓	✗	✗	62 (7.02%)
**EPND**	✓	✓	✗	✗	24 (2.72%)
**IHCC**	✓	✗	✗	✗	58 (6.57%)
**JPND**	✓	✗	✗	✗	174 (19.71%)
**GAAIN**	✓	✓	✓	✓	54 (6.12%)
**Maelstrom**	✓	✗	✗	✗	35 (3.96%)
**UKLLC**	✓	✓	✓	✓	17 (1.93%)
**Other / Web**	✓	✗	✗	✗	10 (1.13%)
**PubMed only**	✓	✗	✗	✗	478 (54.13%)

Abbreviations: ADWB, Alzheimer's disease workbench; AMP‐PD, accelerating medicines partnership program for parkinson's disease; DPAU, dementias platform Australia; DPUK, dementias platform UK; EMIF, european medical information framework; EPND, european platform for neurodegenerative diseases; IHCC, international health coordination centre; JPND, EU joint programme ‐ neurodegenerative disease research; GAAIN, the global Alzheimer's association interactive network; UKLLC, UK longitudinal linkage collaboration.

### 3.3 Data Access

Of the 883 cohorts, 650 (74%) indicated that data were available to third‐party researchers. For 233 (26%) cohorts, third‐party access was either undeclared (n = 224) or declined (n = 9). For 465 cohorts (53%), access was available via direct access to[Table alz70901-tbl-0004] the research team. For 184 cohorts (21%) cohorts, access was via one or more of 34 data repositories, ranging from university repositories (e.g., Vanderbilt Memory and Alzheimer's Center^18^) to national repositories (e.g., the Australian Data Archive^19^). The data repositories varied substantially in the number of datasets and their functionality. Although all 11 dementia‐relevant discovery platforms enabled data discovery, only 7 brokered access agreements between data controllers and third‐party researchers, and only 5 provided full end‐to‐end data management comprising storage, discovery, access brokerage, and analysis: ADWB, Dementias Platform Australia (DPAU),^20^ DPUK,^8^ GAAIN,^11^ and UK Longitudinal Linkage Collaboration (UKLLC).^21^

Data accessibility varied by geographic location. The highest proportion of accessible datasets were found in Europe (80%) and Oceania (78%), whereas lower accessibility was observed in Asia (65%) and the Americas (70%) (Table 2). Multicontinental cohorts indicated the poorest data accessibility (57%).

### 3.4 Data landscape tool

The landscape tool describes cohort, trials, and research register data. At time of writing, it describes 46 research registers, 883 cohorts, and 9687 clinical trials. The tool is updated annually using the methodology we described, incorporating additional cohort studies referenced in newly published papers indexed in PubMed over the preceding year. Future updates are subject to available funding and resources. The tool can be accessed at: https://portal.dementiasplatform.uk/reports/data‐landscape‐report/.

The cohorts section of the landscape tool describes data from 1.8 billion participants, which may be filtered according to study name, country, and continent. Data themes are color‐coded for ease of use. The total number of participants selected is also provided. Documentation levels varied considerably across the data themes, with the highest levels of documentation being for medical history, psychological status, magnetic resonance imaging (MRI) brain imaging, and demographic indicators. Conversely, documentation for variables relating to proteomics, metabolomics, electronic health record linkage, and family disease history was more frequently found to be “undetermined.”

The landscape tool is a convenient means of summarizing data coverage (Table 4). The tool also enables combinations of themes to be selected for more complex searches, identifying relevant cohorts for multi‐cohort analyses. Apart from administrative data, the data themes most frequently covered were participant medical history (82%), lifestyle (78%), and psychological status (75%) (Table 4). These were followed by cognitive status (67%) and sociodemographic factors (60%). Bio‐samples were collected for 45% of cohorts, whereas biomolecular assays are available for 46% of cohorts. Physical measurement (e.g., anthropometry), an epidemiologic and clinical “basic,” was notably obtained for only 41% of cohorts. Imaging and life functionality were assessed for 38% of cohorts, and health care utilization for 26%. Finally, family disease history, linkage to health records, and early adversity data were collected for 19%, 17%, and 16% of cohorts, respectively.

**TABLE 4 alz70901-tbl-0004:** Breadth and completeness of data.

Rank	Data Theme	N (%) cohorts with some data	N (%) participants with some data
1	Administration	883 (100.0)	26,114,129 (100.0)
2	Medical history	723 (81.9)	19,034,116 (72.9)
3	Lifestyle behaviour	688 (77.9)	17,884,021 (68.5)
4	Psychological status	659 (74.6)	10,825,689 (41.5)
5	Cognitive status	600 (68.0)	5,215,625 (20.0)
6	Sociodemographic indicators	536 (60.7)	14,213,632 (54.4)
7	Molecular assays	410 (46.4)	11,613,480 (44.5)
8	Bio‐sample collection	397 (45.0)	10,754,458 (41.2)
9	Physical measures	364 (41.2)	10,369,806 (29.7)
10	Life functionality	339 (38.4)	5,750,424 (22.0)
11	Imaging	337 (38.2)	2,682,883 (10.3)
12	Physical environment	234 (26.5)	10,573,694 (40.5)
13	Healthcare utilisation	233 (26.4)	8,192,155 (31.4)
14	Social environment	212 (24.0)	5,591,642 (21.4)
15	Family disease history	163 (18.5)	7,111,827 (27.2)
16	Linkage data	151 (17.1)	9,760,001 (37.4)
17	Early life adversity	139 (15.7)	5,838,878 (22.4)

The tool also informs an assessment of cohort quality, such as follow‐up rates and timing, data completeness, and attrition. However, this highlights variability in reporting. To estimate the impact of this, we classified metadata documentation levels across cohorts. In this context, documentation refers specifically to the clear presence or absence of variables being collected by a cohort, rather than cases where variable collection status is undetermined. Using a threshold of 80% documentation, ≈24% of cohorts had major documentation gaps, whereas 73% had minor gaps. This distribution informs prioritization for metadata improvement.

Researchers can use the tool's search and filter functions to identify cohorts that meet specific criteria, such as by design, geographic region, and sample size, or those indicating the presence of particular variables of interest, to support tailored cohort selection for diverse research questions.

## 4 DISCUSSION

This study summarizes the cohort study landscape for dementia over the last 50 years. It shows the exponential rise in dementia publications to be due more to cohorts than trials. This reflects substantial investment in new cohort studies, particularly around the millennium, resulting in more than 880 independent datasets.

### 4.1 The landscape

Perhaps the most profound observation from the study is the disparity in regional representation of the global population. Western, educated, industrialized, rich, and democratic (WEIRD) populations are substantially overrepresented. That only 240,389 cohort participants were identified for the continent of Africa (population estimated >1.5 billion) is a call to action, particularly as the greatest growth in dementia incidence is anticipated in the “Global South.” This selection bias constrains generalizability, as it limits data availability on genetic, socioeconomic, and environmental determinants. Greater inclusivity would strengthen the global relevance of dementia research, informing prevention and treatment strategies tailored to diverse populations while addressing health disparities worldwide.^22^

The roots of this imbalance are complex. Systemic disparities in research funding, infrastructure, and data governance across regions contribute to the underrepresentation of low‐ and middle‐income countries (LMICs). Ethical, logistical, and regulatory challenges further limit the feasibility of conducting large‐scale cohort studies.^15^, ^23^ Addressing these structural barriers is essential for a more equitable global research landscape. International funding mechanisms prioritizing underrepresented regions and equitable partnerships promoting local leadership and sustainability are needed to support LMIC initiatives.^23^ Such efforts would not only improve representation but also enhance the scientific value and global relevance of dementia research. Poor data access in multicontinental cohorts likely reflects the complexity of data sharing across jurisdictions, including diverse legal frameworks, and managing governance, ownership, and compliance.

Other characteristics of the cohort landscape are less stark. Gender representation is balanced, and age distributions reflect cohort design and trends in chronic disease research. That fewer cohorts have been commissioned since the millennium may be a reporting artifact, or may instead reflect a general enthusiasm for cohorts at the turn of the century.

### 4.2 Scientific opportunity

“The cohort study can be considered the direct analogue of the experiment, in the sense that it is the investigator who selects subjects to observe, and who classifies these subjects according to exposures.”^24^ In this sense, albeit with its own execution challenges, the cohort provides a uniquely comprehensive and versatile design by which realistically complex and emerging questions can be addressed.

At the investigator level, the versatility of cohort data is widely recognized, as the number of publications using cohort data is increasing exponentially. The emergence of generic, large‐scale, in‐depth population studies such as UK Biobank, All of Us, and Our Future Health suggests this trend will continue.^25^, ^26^, ^27^

For data discovery, scientific institutions have been slow to respond. Search engines favor findings and authorship but are ill suited for discovering datasets. This limitation extends beyond PubMed, reflecting a community‐level bias. For example, the “Strengthening the Reporting of Observational Studies in Epidemiology (STROBE)” guidelines,^28^ focus on reporting rigor, not discovery or reuse. This problem has been recognized by several journals, notably the *International Journal of Epidemiology*, with its introduction of Cohort Profiles.^29^ However, the detail and capacity possible in this solution is limited.

Metadata quality varied considerably in detail and format. Although cohort research teams are often required to make their data available to third parties, they are rarely sufficiently resourced to do so. As a result, metadata may suffice internally but often remain opaque to third parties.

### 4.3 Limitations

Using a single search engine to identify datasets would be a more serious limitation if the goal was to identify findings. We consider it unlikely that many further independent datasets would have been found by using multiple search engines, given that we also searched relevant data platforms.

Nevertheless, the number of datasets identified cannot be considered exhaustive. Reliance on publicly available metadata introduces selection bias, favoring cohorts that are better resourced or more transparent in their reporting. This will likely result in the underrepresentation of smaller, less visible, and possibly more scientifically informative studies. However, the review represents best estimates derived from limited data from multiple sources. These numbers indicate general levels of activity and providing an overview of the data collected and its availability. Many of the characteristics of the cohort ecosystem are apparent, and the trends described here may be considered useful in directing research activity and funding programs.

The granularity of the metadata is high level. This was constrained by variability in the detail of publicly available information. Although greater detail was available for some cohorts, a broader and more inclusive strategy was adopted for discovery and comparison.

These limitations are a function of our literature‐based metadata collection strategy. An alternative would be to invite cohort research teams to submit study details, as used for mental health in the Atlas of Longitudinal Datasets^30^ and for birth cohorts in Birthcohorts.net.^31^ Although efficient for collating minimal metadata, this approach places a reporting burden on research teams if variable‐level metadata are requested. For this study, the strategy was to provide a more detailed overview of dementia cohort data that the community can build on, both through improving precision and proposing further datasets. To this end, we encourage research teams to improve the accuracy and range of metadata in the online landscape tool.

### 4.4 Looking ahead

In this article, we have summarized the dementia cohort data landscape, highlighting challenges in structuring and organizing these data. These stem from: (1) a lack of data, metadata, and reporting standards, and (2) structural issues (incentives and transaction costs) around standard development and adoption. Arguably, these apply to cohort data generally but it is helpful to use dementia as an exemplar.

The growing pool of cohort data presents substantial scientific opportunities for reuse, offering considerable cost and time savings, particularly from longitudinal follow‐up data.^32^ Realizing these benefits can be accelerated by standardized data, metadata, and reporting. Here we used C‐Surv as the data, metadata standard, but other models are available. However, a broader stakeholder‐wide initiative involving researchers, funders, and editors would facilitate standards development, and wider adoption. Requiring persistent identifiers for publicly funded datasets for funders and editors would incentivize the development of standards. Established standards provide a basis for tool development. The use of artificial intelligence to reduce the transaction costs of improving discoverability and accessibility will likely be transformative.

A guiding principle of strategically organized cohort data is maintaining public trust. As datasets become more sensitive and complex, safeguards protecting consent criteria and participant identity must strengthen. End‐to‐end data management solutions provide trust‐by‐design, standard and streamlined access pathways. Although challenging to build and maintain to ever‐increasing security standards and computational requirements, there is no obvious alternative that will satisfy public and funder expectations for the safe and efficient stewardship of publicly funded data. These data environments appear in various guises, including trusted research environments (TREs), data enclaves, and secure data environments. Nevertheless, these share common characteristics, including privacy protection, access brokerage, standard legal agreements, no external data transfer, and output screening to ensure non‐identifiability. It would be unsurprising if the use of secure data repositories was made mandatory by funders and journals within the next 5 years.

TREs also bring operational advantages. Although building and maintaining TREs for individual studies is relatively expensive, they represent cost‐savings when serving multiple studies by providing a nexus for standardizing and streamlining data/metadata models, data management procedures, and legal agreements. These simple procedures reduce duplication and fragmentation within the data landscape and create an environment where enhancing research data with real‐world clinical data can be routinely achieved. Using data access as an example from the 650 cohorts allowing access, 184 (28%) indicated this was available via a data repository. At a conservative estimate, this suggests that more than 450 bespoke access pathways are operating. Few researchers have the time or resource to navigate such a labyrinthine environment. TREs offers an efficient solution.

For making cohort data securely available globally, federation across data platforms is probably the only solution capable of addressing complex sovereignty and governance issues. The need for aligning datasets for federation provides a catalyst for ongoing standards development. To support this, models such as federated data governance and harmonized ethics protocols help bridge coordination gaps and enable scalable collaboration.

### 4.5 Conclusions

This analysis provides insights into the dementia research landscape, identifying gaps and barriers that impede the repurposing of existing datasets. Addressing these issues, through improved data and metadata reporting, standardized access, and broader inclusivity, will maximize the utility of existing resources. By leveraging dementia research as an exemplar, this article informs strategies to strengthen the broader health cohort infrastructure, driving innovation and efficiency in chronic disease research.

An international register of dementia‐relevant cohorts would incentivize the development of data, metadata, and reporting standards. It would also provide a single authoritative source for data discovery fulfilling a role comparable to the NIH clinical trials register.^33^ This would enable and encourage greater data sharing and access. This landscape tool is a step toward that goal.

## CONFLICT OF INTEREST STATEMENT

The authors declare no conflict of interest. Any author disclosures are available in the Supporting Information.

## CONSENT STATEMENT

The research undertaken for this article involved no human subjects.

## Supporting information



Supporting Information
